# Inhibition of *acn-1* , an ACE ortholog, reduces intestinal GST-4 expression in *Caenorhabditis elegans*

**DOI:** 10.17912/micropub.biology.001682

**Published:** 2025-10-09

**Authors:** Josiah King, Kristopher Schmidt

**Affiliations:** 1 Department of Biomedicine, Eastern Mennonite University, Harrisonburg, Virginia, United States; 2 Department of Biology & Chemistry, Eastern Mennonite University, Harrisonburg, Virginia, United States

## Abstract

We investigated the role of *acn-1* , the *C. elegans* ortholog of human ACE and ACE2, on the expression of GST-4 , an antioxidant molecule produced in response to oxidative stress. *acn-1* RNAi exposure significantly reduced GST-4 reporter expression after 24 and 48 hours. The difference in *gst-4* expression appears most prevalent in the intestine. Our findings suggest that *acn-1* function positively influences *gst-4* expression in *C. elegans* .

**Figure 1. Basal GST-4 expression diminishes when acn-1 is inhibited:**
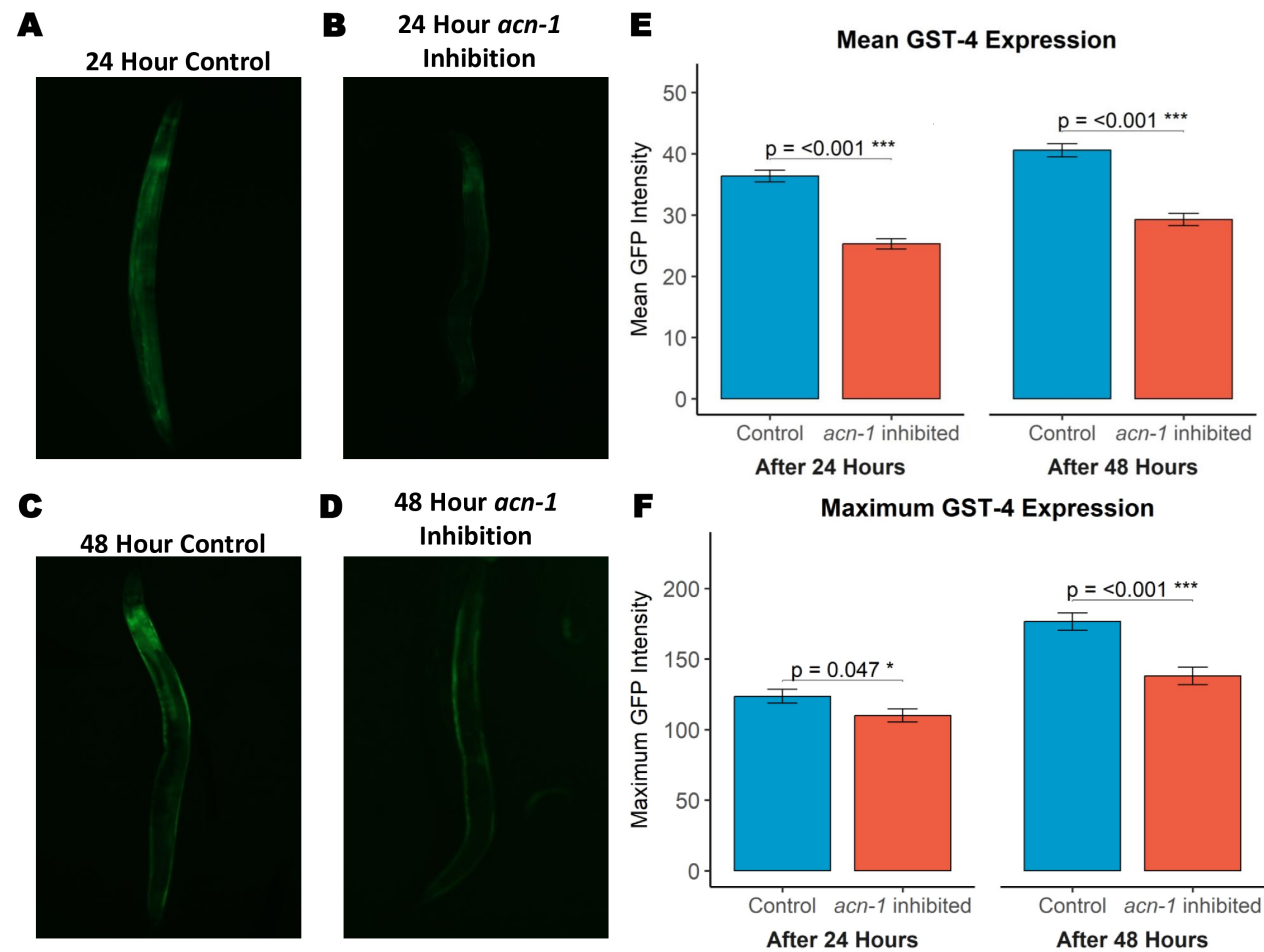
(A) Representative images showing GFP expression in worms exposed to *E. coli* containing an empty vector for 24 hours. Worms in this group appear to express high intestinal GFP. (B) Representative images showing GFP expression in worms exposed to *acn-1* RNAi for 24 hours. (C) Representative images showing GFP expression in worms exposed to *E. coli* containing an empty vector for 48 hours. Worms in this group appear to express high intestinal GFP. (D) Representative images showing GFP expression in worms exposed to *acn-1* RNAi for 48 hours. Worms in this group often lacked detectable GFP expression in the intestine region. (E) Mean GFP intensity throughout the whole body after 24 hours (p < 1e ^-14^ ; N=80 control, N=82 *acn-1* RNAi, ± SEM across 3 experiments) and 48 hours (Mean p < 1e ^-11^ ; N=76 control, N=72 *acn-1* RNAi, ± SEM across 2 experiments). F. Maximum GFP intensity throughout the whole body after 24 hours (p < 4.68e ^-02^ ; N=80 control, N=82 *acn-1* RNAi, ± SEM across 3 experiments) and 48 hours (p < 6.10e ^-06^ ; N=76 control, N=72 *acn-1* RNAi, ± SEM across 2 experiments).

## Description

While previous studies have linked *acn-1* to life extension and stress resistance in *C. elegans* , the mechanism by which this is done remains unclear. Inhibition of *acn-1* is reported to increase lifespan and oxidative stress resistance. There is a significant gap in scientific literature in regard to interactions between *acn-1* and regulatory reporters, like GST-4 . GST-4 is an antioxidant that is released in response to oxidative stress (Pohl et al., 2019).

Our quantitative image analysis revealed a drastic decrease in GFP mean fluorescence after 24 hours (p < 1e ^-14^ ; N=80 control, N=82 *acn-1* RNAi, 3 experiments) and an even larger difference after 48 hours (p < 1e ^-11^ ; N=76 control, N=72 *acn-1* RNAi, 2 experiments) (Figure 1). To determine whether the signal reduction was more localized or uniform, we also analyzed the maximum intensities of fluorescence. A similar reduction was observed after both 24 hours (p < 4.68e ^-02^ ) and 48 hours (p < 6.10e ^-06^ ), with the brightest signals frequently being located in the caudal intestine region.

Our experimental results reveal that when *acn-1* is inhibited, *gst-4* is expressed less. This indicates that *acn-1* regulates *gst-4* expression, but the pathways and interactions through which it does so are still not clear. Despite being frequently used as a marker of oxidative stress, *gst-4* can be expressed through multiple pathways that are not all exclusively induced by oxidative challenge (Detienne et al., 2016). While the mechanism by which *acn-1* regulates *gst-4* is unclear, literature indicates life extension mediated by *acn-1* requires the transcription factor *daf-16* . *gst-4* is known as a target gene of *daf-16* . However, based on current evidence, *acn-1* does not appear to impact the nuclear localization of DAF-16 (Kumar et al., 2016). This suggests that *acn-1* either affects specific target genes of *daf-16* or works in parallel with *daf-16* . Because of the compensatory nature of detoxification molecules (Back et al., 2010), we suspect that the decreased *gst-4* expression during *acn-1* inhibition reflects a redistribution of stress response gene expression where *gst-4* is reduced and offset by the increase of other *daf-16* target genes like *gst-10* , which increases lifespan when overexpressed (Shields et al., 2021).

In summary, inhibition of *acn-1* decreases *gst-4* expression, and this suggests a positive regulatory role. This reduction in *gst-4* may reflect a compensatory shift in gene expression where other *daf-16* regulated genes associated with life extension are upregulated (Back et al., 2010; Park et al., 2009). *acn-1* shows some homology with human ACE, and ACE inhibitors affect aging in various organisms (Brar et al., 2018; Egan et al., 2022). These findings may point to a conserved mechanism worth further investigation. Despite being homologous to human ACE at the sequence levelc, however, ACN-1 lacks several histidine residues that are necessary for the peptide-cleaving functions of ACE. ACN-1 plays an essential yet unclear role in nematode larval molting, a functionality that is unrelated to the role of human ACE in the renin-angiotensin system (Egan et al., 2022). Due to the functional differences, these results should not be interpreted as directly applicable to human ACE functions and human ACE inhibitors. Further investigation in mammalian models is required to establish any potential parallels.

## Methods

**RNAi inhibition of *acn-1***

A CL2166 strain of *C. elegans* with *gst-4 p* ::GFP reporter was ordered from the University of Minnesota's Caenorhabditis Genetics Center ( *Genotype: dvls19 III. 2024* ). Samples of *E. coli* containing *acn-1* RNAi clones from the Ahringer RNAi library were received from the Kornfeld Lab (Egan et al., 2024). Gravid *C. elegans* populations were synchronized using a bleaching protocol (Stiernagle, 2006). The resulting eggs were allowed to hatch on NGM plates with no food for 30 hours. Simultaneously, *E. coli* was induced for 30 hours with 1 mM IPTG. The *E. coli* containing *acn-1* was grown alongside *E. coli* containing pPD129.36, an empty vector used for control. After 30 hours, the hatched L1s were washed onto NGM plates containing *E. coli* ( OP50-1 ). After 25 hours, the now synchronized L3-L4s were washed to the IPTG NGM plates containing RNAi. Half of the worms were transferred to plates of *E. coli* containing *acn-1* RNAi, and the other half were transferred to plates of *E. coli* containing pPD129.36.

**Fluorescent assessment of GST-4 expression**

*gst-4* expression was assessed under the Leica M205 FCA fluorescence stereo microscope. *C. elegans* were imaged under NaN₃ (20 mM) on NGM plates. A set of worms was analyzed after 24 hours of *acn-1* RNAi exposure (N=80 control, N=82 *acn-1* inhibited), and another set was analyzed after 48 hours of RNAi exposure (N=76 control and N=72 *acn-1* inhibited). 24-hour procedures were replicated on 3 different days, and 48-hour procedures were replicated on 2 different days. ImageJ was used to analyze all the images by first splitting the images into separate color channels and selecting out only green fluorescence. The entirety of each worm body was selected using the polygon tool. Then the fluorescent intensity was assessed on a normalized scale of 0-255. Finally, the mean and maximum fluorescent intensity were measured and analyzed statistically using Pearson's t-test.

## Reagents

**Table:** 

**Strain**	**Genotype**	**Available From**
CL2166	*dvIs19 [ gst-4 p::GFP::NLS]* III	CGC ( Caenorhabditis Genetics Center)
**Bacteria/RNAi constructs**	***Description***	**Available From**
OP50-1	Wild type used as a food source.	CGC
*E. coli* HT115 (DE3) + *acn-1* RNAi	* E. coli that contains dsRNA plasmid targeting acn-1*	Kornfeld Lab, Washington University in St. Louis
*E. coli* HT115 (DE3) + pPD129.36	*Empty vector used as RNAi control*	Addgene or CGC RNAi library
**Plasmid**	***Genotype***	**Description**
pPD129.36	*Empty vector*	Standard L4440-based RNAi control plasmid used for feeding RNAi.
**Tool**	***Purpose***	**Source**
ImageJ	*Fluorescence intensity analysis*	NIH
UCSC Genome Browser	*RNA-seq visualization*	UCSC Genomics Institute
NCBI BioProject	*RNA-seq data acquisition*	NCBI
